# Developing a healthcare worker psychological preparedness support programme for the COVID-19 outbreak

**DOI:** 10.4102/sajpsychiatry.v28i0.1665

**Published:** 2022-03-10

**Authors:** Zukiswa Zingela, Stephan van Wyk, Aletta Bronkhorst, Carmenita Groves

**Affiliations:** 1 Executive Dean’s Office, Nelson Mandela University, Gqeberha, South Africa; 2 Department of Psychiatry and Behavioural Sciences, Walter Sisulu University, Mthatha, South Africa; 3 Mbulawa Mental Unit, Dora Nginza Hospital, Gqeberha, South Africa

**Keywords:** COVID-19, mental health, health worker, psychological, support

## Abstract

**Background:**

The coronavirus disease 2019 (COVID-19) outbreak caused worldwide disruptions to healthcare systems. The emerging evidence indicates that mental health problems have consequently become an occupational hazard in frontline healthcare workers.

**Aim:**

We aimed to develop a psychological preparedness training (PPT) programme to support frontline health workers in three resource-limited hospitals in South Africa dealing with the COVID-19 outbreak and to evaluate its effectiveness using an audit tool. We established a theoretical framework and goals for a psychological preparedness programme to support healthcare workers at the study sites.

**Setting:**

Data were collected at the Dora Nginza Hospital, Nelson Mandela Academic Hospital and Elizabeth Donkin Hospital.

**Methods:**

We employed an observational, descriptive, and cross-sectional design. A group psychological intervention was developed and implemented at the three sites in South Africa, from mid-April 2020 over 20 weeks. We collected data using an audit tool to measure healthcare workers’ perceptions of the outbreak before and after the intervention. We analysed the data to test for a statistically significant difference between the pre-intervention and post-intervention audit tools.

**Results:**

We supported 761 healthcare workers during the 20 weeks of the programme. Statistical analysis showed a significant positive change from pre- to post-intervention measures in perceptions of health worker about the outbreak, their anxiety associated with the outbreak, their ability to control reactions to stress and the perception of their ability to support others. Feedback comments indicated that the programme was beneficial for the majority of those who attended.

**Conclusion:**

Health workers who attended the programme reported improvement in stress levels and in perceptions about their ability to cope with the outbreak, as well as in their perceptions of being able to support others.

## Introduction

The World Health Organization (WHO) declared the coronavirus disease 2019 (COVID-19) outbreak a pandemic on 11 March 2020.^1^ South Africa (SA) reported the first positive case for the causative coronavirus on 05 March 2020.^2^

The negative effect of the COVID-19 outbreak on healthcare systems was described in countries, such as China, where the outbreak emanated, Italy, Spain, and the United States of America (US).^3^ The resultant mental health burden was also reported in a survey of 1257 healthcare workers in China, with up to 50% of those surveyed having significant disturbances in their mental health, such as anxiety, depression, insomnia and distress.^4^ We developed a group psychological preparedness intervention programme in a tertiary service level general hospital in the Eastern Cape (EC) province to enhance the coping skills of health workers and mitigate the potential negative effects of the pandemic on their mental health.

The EC, with close to 6.7 million, people ranked fourth in terms of provincial COVID-19 cases at the onset of the outbreak.^5^ At that point, EC had 13 psychiatrists, 15 clinical psychologists, four registered counsellors and 18 mental health social workers in the public sector. The province thus lacked capacity to provide individual interventions for healthcare workers requiring help from the public sector for emergent mental health problems because of the outbreak. These limitations necessitated an innovative approach for delivering mental healthcare services for healthcare workers.

The concept of psychological preparedness for the outbreak was modelled based on evidence for the usefulness of psychological preparedness interventions in communities facing natural disasters.^6,7,8,9^ Some studies have shown interventions targeted at psychological preparedness are a useful approach to support such communities.^6,8,9^ Roudini et al. defined three important elements of psychological preparedness.^9^ These include a state of awareness and expectation of one’s psychological reactions to a disaster, having the ability to identify stress-related emotions and thoughts generated by the disaster, and being able to deal with those negative emotions and thoughts in an adaptive way that enhances coping. Important components of psychological preparedness for communities and individuals are similar across different disasters.^10^ Preparedness also includes management of, or coping with one’s thoughts, feelings and reactions to the disaster.

Psychological preparedness is reported to enhance logical thinking and problem solving when the disaster hits.^6,8^ Preparing individuals psychologically for disasters may enhance the use of adaptive coping strategies and help them develop a resilient response to the event, and possibly foster long-term resilience.^6^ Disaster preparedness strategies must meet the mental health and psychosocial needs of the community for which they are designed. Mental health preparedness may also be a helpful strategy for protecting individuals from the negative psychological impact arising from unexpected disasters.^6,8^ There is a positive connection between disaster preparedness and mental health, and the probability of a mental disorder following disasters is linked to an absence of preparedness.^10^

We approached the outbreak in a similar way to these recommendations for natural disasters and worked from the premise that psychological preparedness could mitigate the probability of a mental disorder following health workers’ exposure to health systems stressed by COVID-19. We combined this with an approach for mass trauma by adopting goals for the programme based on a consensus paper that reported five essential elements for mass trauma intervention.^11^ These principles of intervention for dealing with the anticipated fallout associated with traumatic events are the following: to promote a sense of safety, a sense of calm, a sense of self– and collective efficacy, connectedness and a sense of hope.

### Aims

- To develop a group psychological preparedness training (PPT) programme to support health workers in three hospitals dealing with the COVID-19 outbreak.
- To evaluate the impact of the programme on healthcare workers using an evaluation tool derived from an audit tool to measure health workers’ perceptions of and reactions to the outbreak, before and after the training.

### Objectives

- To develop a group psychological preparedness programme to support healthcare workers during the outbreak based on psychological principles for cognitive behavioural therapy and mass trauma interventions.
- To implement the support programme for healthcare workers in three health institutions in the EC.
- To audit the programme and evaluate its effectiveness through the application of a ‘before and after’ intervention evaluation tool.

## Research method and study design

We conducted an observational, descriptive study using a quantitative and exploratory qualitative approach to examine the emotive and cognitive experience of healthcare workers with respect to the COVID-19 outbreak, before and after attending a specially designed support programme. We first audited the programme from mid-April 2020 to the end of May 2020 (6 weeks) using an audit tool in order to determine utility at the study sites. We then used an amended, shorter evaluation tool guided by the audit results, to assess health worker feedback from June 2020 to the end of August 2020 (14 weeks). We collected data on two types of feedback, which were feedback on the programme and feedback on how healthcare workers perceived the outbreak, and their ability to cope before attending the programme and after completing the programme.

### Setting

Study sites were three hospitals in the EC, SA, the first being the Nelson Mandela Academic Hospital Complex, comprising a tertiary and regional general hospital situated in Mthatha. The second was the Dora Nginza Hospital (DNH), a regional general hospital, and the third was Elizabeth Donkin Hospital, a psychiatric hospital, both in Nelson Mandela Bay Metropolitan Municipality (NMBMM). We chose these three sites because of the willingness of their multidisciplinary mental health teams to facilitate the programme.

The Nelson Mandela Academic Hospital Complex in Mthatha is in the OR Tambo district and serves a mostly rural population of over 3 million in the eastern part of the EC province.^5^ Dora Nginza Hospital and Elizabeth Donkin Hospital are both in the western part of the EC and serve a mostly urban population of 1.2 million people.^5^ The three hospitals accept referrals from health institutions that provide primary health services, including mental health services, to the general population.

### Participants

Attendance was voluntary, and in the majority of cases, attendees requested the sessions instead of waiting to be referred by their line managers. Some attendees also reported that word-of-mouth from colleagues who felt they had benefited from the programme influenced their decision to self-refer. Interested healthcare workers in the three hospitals were invited to participate through information disseminated via worker WhatsApp groups, heads of departments, and staff meetings. Written information about the programme and how to access it was also provided (see Appendix 1). A telephonic or WhatsApp booking system was used to arrange attendance at group sessions that were run up to three times a week, each 60–90 min long. Lead facilitators were identified at each site to manage the booking system. Any employee at the three hospitals could access the programme.

#### Inclusion criteria

Any healthcare worker who wished to attend during day shift hours was included.

#### Exclusion criteria

Healthcare workers who had flu-like symptoms, even if they had not tested for COVID-19 yet or those who were awaiting COVID-19 test results, were excluded from attending the sessions.

### Sampling

The number of healthcare workers at the three sites was just over 3000, with approximately 80% anticipated to perform direct COVID-19 outbreak-related duties at the point of starting the programme. The margin of error or confidence interval (CI) was set at 95%, and the standard deviation (SD) was set at 0.05. In order to determine the total sample size required, we utilised the formula:


$$n=N{\text{/}(1+\text{Ne}}^{2})$$ [Eqn 1]

yielding a minimum sample size of 343. We added 20% (69) to account for possible data entry errors and non-responses. The appropriate total sample size for valid findings for the audit and evaluation of the programme was 412.

### Assessment tools

The audit tool for the programme was developed guided by Zulch and McLennan and Marques, papers on psychological preparedness for disasters in terms of content.^6,8^ The audit tool by Zulch was developed and validated for assessing preparedness for extreme weather, specifically cyclones.^6^ For us to be able to use the Zulch audit tool as a guide, we approached the outbreak as a major stressor that health workers would have to face and endure with the propensity to cause mass trauma. The tool, however, was not validated for use in outbreaks like COVID-19, and it was not designed to measure the success of an intervention. Essential elements of the audit tool focused on the emotive and cognitive experience of the COVID-19 outbreak. There was also a feedback comment section added to enable the health worker to provide information on how they felt they were coping with the outbreak before and after attending the support programme. For the first six weeks, this information was collected via the longer audit tool with 26 questions based on the original Zulch 26 item scale, and with all the questions adapted to focus on the outbreak instead cyclones.

We then further adapted this to a shorter evaluation tool with 10 questions, which we used over a 14-week period. The 10 questions were chosen based on the facilitator consensus about which 10 items were considered crucial in the 26-item audit tool (see Appendices 2 and 3) for application to the COVID-19 outbreak. Time constraints and observed worker weariness, especially at the beginning of the sessions, also influenced the shortened evaluation tool. Healthcare workers were working under extreme pressure with limited time available, and many found that filling in a long pre-intervention evaluation tool was tedious.

### Variables

Both the audit and evaluation tools allowed attendees to feedback their perceptions of the sessions in a section included for ‘comments’. This was to provide a free flow of thoughts about the programme immediately after the session. We further collected qualitative data on participant’s perceptions and experience of the outbreak and the perceived effect that the outbreak had on them and their lives. Participants were requested to write down feelings, thoughts and behaviours which they thought they were experiencing as a result of the pandemic. The statement posed to the healthcare workers was: ‘since experiencing the outbreak, describe how your thoughts, feelings and behaviour has been affected’. The evaluation tool included survey questions focused on anxiety about and coping with the outbreak. Participants were supported and guided to identify and express feelings of distress and then practice the various coping strategies to manage the distress, which included basic relaxation techniques, mindfulness techniques and cognitive strategies.

### Costs and resources for developing and implementing the programme

There were minimal costs associated with the development and implementation of the programme. The facilitators were EC Department of Health employees working at the three health facilities, with venues within the health institutions, allowing for physical distancing as recommended by the South African COVID-19 regulations that were in effect at the time.^12^ Each session was run by two to three facilitators drawn from a multidisciplinary team of psychiatrists, psychiatrist-in-training, medical officers, clinical psychologists, registered counsellors, social workers and professional psychiatric nurses who were employed in the Department of Psychiatry across the three hospitals. The facilitating team in the three hospitals included four psychiatrists, 14 psychiatry registrars (residents), three medical officers, five mental health social workers, five clinical psychologists and one registered counsellor. The recommendation was for a minimum number of two facilitators with a maximum of three per session.

### Approach to implementation

The delivery of the programme was performed in three parts to address the five principles highlighted for mass trauma interventions: mind care, relaxation techniques and team care. The attendees were guided to:

- identify thoughts, feelings and behaviours linked to the COVID-19 outbreak;
- identify which of their thoughts, feelings and behaviour could undermine their ability to cope with the outbreak, and how to develop logical counterarguments to deal with these;
- formulate and reinforce thoughts to generate feelings and behaviour that could enhance their sense of coping, to promote calm and self-efficacy;
- identify and reinforce team successes and strengths, to promote team efficacy and connectedness;
- use available evidence and facts to refocus on positive news and ‘successes’ in the fight against the outbreak to reignite hope.

Before facilitating sessions for other health workers, all facilitators were first taken through the programme by the lead facilitator who designed the programme. This was carried out over 60–75 min. The goal was to train facilitators on how to facilitate a session. They reported additional benefits, finding that going through the support and training session before facilitating sessions for others also enabled them to become more aware of their anxieties and fears about the outbreak and provided an opportunity to practice how to manage these more effectively.

### Running of the groups

Health workers could attend one group session at a time. They were then encouraged to consolidate the new skills and information learned and were informed they could return at a later date if they so wished, as long as they pre-booked for a session to enable a reasonable control of the number of attendees per session. Each group session had three components to it, delivered over 60–90 min. All attendees received the same programme. The period for each group was planned for 60 min but some attendees were more interactive and tended to express themselves more which would influence the actual length of the session. There were no more than a handful who attended again, and they were mainly focused on reinforcing what they had learnt about relaxation and mindfulness techniques. They self-identified as returnees and did fill in another audit tool.

The content of the group sessions was the following: it started with a 10-min introductory part where facilitators and attendees introduced themselves to each other, a short explanation of the goals of the sessions linked to the five principles of intervention for mass trauma and why and how these could be applied to the outbreak, the structure of the programme for the session, the role of the audit or evaluation tools as well as consent issues. Facilitators then guided the attendees over 30 min through the ‘Mind Care’ part of the session. This involved a process where attendees identified and wrote down the effect of the outbreak on their thoughts, feelings and behaviours. Attendees would then be guided through an interactive process to identify which of their reactions enhanced their ability to cope with the outbreak and which impacted negatively on their ability to cope. The Mind Care was often accompanied by emotions of distress expressed as anger, anxiety, fear, worry and other feelings, which attendees reported as overwhelming. Facilitators were able to manage the distress because the Mind Care was followed by a 20-min interactive and practical process of teaching attendees relaxation and mindfulness techniques, as well as basic cognitive and behavioural strategies of targeting the thoughts and reactions they flagged as unhelpful to coping. Attendees were guided to identify reactions that enhanced coping and were encouraged to share tips on what worked with each other. The last couple of minutes was dedicated to tips on ‘Team Care’, which focused on how attendees could support each other at work to promote team efficacy, enhance team morale and strengthen cooperation.

### Bias and validity

Potential sources of bias were as follows:

1. Sampling bias because of the exclusion of staff who did not have access to a phone that could send messages via WhatsApp. This number was considered negligible because of the growing use of WhatsApp as a communication and access to information tool in the clinical health space in the region.^13,14^
2. The use of a written audit tool in English, which relied on translation for those who might have needed it. This number was also considered negligible since most of the healthcare workers held jobs whose minimum requirement was high school education and English is the language of instruction in South African high schools.
3. The evaluation tools had not been validated for use in this setting nor had it been validated for assessing the effectiveness of the group intervention programme, which may affect the applicability and generalisability of the results.

### Statistical methods and data analysis

#### Quantitative data analysis

We summarised and analysed the quantitative data from the audit and evaluation tools using categorical variables and frequency tables. Because of the ordinal nature of the data, we used a nonparametric test, the Mann–Whitney *U* test, to test whether there was a statistically significant difference between the 26-items of the audit tool and 10-items of the evaluation tool, within the pre- and post-intervention surveys.

We used the independent sample *t*-test to determine whether the average factor score differed significantly from the pre- to post-survey items. The independent sample *t*-test was used instead of the paired sample *t*-test because the responses were not all matched according to respondent code, and there were a different number of responses for the ‘pre’ and ‘post’ tools submitted. Therefore, we determined the differences based on the independent groups. We performed an exploratory factor analysis (EFA) for both the 26-item and 10-item tools.

#### Qualitative analysis

The lead investigator and two research assistants evaluated the healthcare worker comments and feedback on their perceptions and experience of the support programme. This was performed during four consensus meetings to systematically organise the qualitative data according to linguistic expression and content analysis as guided by the consolidated criteria for reporting qualitative research (COREQ).^15^ We collected all comments received from healthcare workers who attended the sessions onto a spreadsheet. We then utilised Braun and Clarke’s analysis for qualitative data to identify themes emanating from the comments.^16^ We identified word repetition, similarities and keywords, which were then grouped. The three assessors worked independently initially, and then engaged in the four consensus sessions to compare thematic categories and engage further to reach a consensus about grouping of the main themes. The process of grouping was repeated and refined during the consensus meetings until no further similarities or repetition of words could be identified.

The evaluation and analysis of documented thoughts, feelings and behaviours stemming from the outbreak falls outside the purview of this study.

### Ethical considerations

The chair of the ethics committee of the Walter Sisulu University was approached regarding the question of whether ethical approval would be required for use of the audit tool to assess the group intervention programme during the early stages of implementation and provide information on its applicability and appropriateness in this setting. The feedback received was that because the programme mainly had a training element to it, and the audit process would not change the components or delivery of the planned programme, therefore it could be audited without ethical approval for a limited period to provide answers on the need for review of the process for programme evaluation. Attendees had to be informed of the voluntary nature of filling in the audit tool and their right to withdraw their feedback information from the overall analysis and were also required to give consent for inclusion of their individual audit tool in the general analysis. Attendees were also informed that audit results would form part of a feedback process to other healthcare workers in the mental health service field to assist the learning process of how to support health workers during a pandemic. Finally, any data collected after this limited time period, however, would require ethical approval. Ethical approval (protocol number: 027/2020) was granted by the Walter Sisulu University Human Research Ethics Committee for evaluation of the programme following the audit, using the amended 10-item evaluation tool.

Verbal consent for completing and submitting the audit tool (first 6 weeks) and evaluation tool (subsequent 14 weeks) was obtained from the attendees at the beginning of each session. We provided an explanation about the role of the tools in evaluating the utility of the programme for attendees and an opportunity for questions and concerns about the tools to be addressed before the session. The audit and evaluation tools were anonymised to delink them from any identifying details, and attendees who did not wish for their data to be included in the analysis had the option of withholding the tools for their private use only.

## Results

### Quantitative

Up to 761 healthcare workers attended the programme , which was facilitated by a multidisciplinary team of 32 across the three sites. This equated to a ratio of one facilitator for every 23.8 healthcare workers who attended the sessions. Of the 761 health workers who attended, 192 filled in and returned the pre-intervention surveys and 760 filled in the post-intervention surveys. The 192 pre-intervention surveys submitted consisted of 86 of the 26-item audit tool and 106 of the 10-item evaluation tool. The 760 post-intervention surveys consisted of 105 of the 26-item and 655 of the 10-item tool.

### Qualitative

There were two categories of qualitative variables collected. The first was on healthcare workers’ perceptions about the support programme. Up to 358 post-intervention comments were received. The second category was collected during the 14 weeks following the audit of the programme and was on perceptions of healthcare workers about the outbreak. A total of 351 descriptions of thoughts, 128 descriptions of feelings and 327 descriptions of behaviours that healthcare workers associated with the advent of the outbreak, were collected. The report on the qualitative data and analysis of this study will be limited to healthcare workers’ perceptions about the support programme. Three main themes that emerged from the feedback received indicated that the programme was (1) helpful and good (or excellent) in 127 (35.5%) of the 358 comments, (2) informative in 80 (57.8%), and (3) enabled attendees to feel that they can manage outbreak-related and other stress and cope better with it in 117 (32.7%) of the 358 comments. This means up to 68.2% of health workers who attended the programme found it helpful and useful for managing stress, and up to 90.5% found it informative and useful for managing stress. The remaining 34 (9.5%) comments did not fit into any unified theme.

During facilitator discussions about the programme, facilitators indicated that starting the facilitation process by first being taken through the programme, and then going on to implement it for other fellow healthcare workers, enhanced their ability to cope with their fears and anxieties about the outbreak. It also enhanced teamwork and levels of cooperation and collaboration within the facilitating teams.

## Results analysis

### Quantitative data analysis

The data were initially cleaned to remove any responses that had missing data within the survey questions. We did not exclude the missing data within the profession question because it was not essential to the audit. Initially, we grouped the data according to the respondent code for pre-PPT and post-PPT data. However, because they were not all matched because of anonymity, we made use of the independent sample tests to show whether there was a significant difference between pre- and post-intervention.

#### The 26-item audit

Question 1 (Q1) in the audit was about assessing the likelihood of the outbreak ‘reaching us’, and Question 2 (Q2) was about monitoring news bulletins about the outbreak. Questions 3 (Q3) to 26 (Q26) were about knowledge of, training for, coping with, and reactions to the outbreak. There were also items that asked about the ability to calm oneself and others during an upsurge of infections. According to the results, items Q3 – Q26 showed a significant difference from pre- to post-intervention, where according to the Sum of Ranks, the post-surveys showed greater (more positive) scores than the pre-surveys. Q1 and Q2 did not show any statistically significant difference in the responses in both pre- and post-intervention responses.

#### Exploratory factor and reliability analysis

Regarding the EFA, the Kaiser–Meyer–Olkin measure of sampling adequacy (KMO–MSA) value of 0.92 and significant Bartlett’s test of sphericity indicated that the data were appropriate for EFA. The variance extracted was also 52.61%, which is within the acceptable range. The factor loading cut-off was set to 0.40. Items Q1, Q2, Q10 and Q25 did not load significantly on any factors, whilst the remaining 22 items loaded onto three factors, which were all deemed to be reliable.

#### Factor scores (descriptive)

The factor scores per survey, according to pre- and post-intervention data, show that the means differ according to whether they were taken pre- or post-intervention.

#### The independent t-test for the 26-item survey

Q26_Factor_1, showed that there was a statistically significant difference pre- (*M* = 2.77, standard deviation [s.d.] = 0.66) and post-intervention (*M* = 3.57, s.d. = 0.44) (*t* = –9.7, *df* = 144.22, *p* < 0.001). In other words, each factor showed a significant difference in the average factor score for pre- and post-audit data. Figure 1 illustrates the differences between the pre- and post-questions, which made up the first 10 of the 26-item audit. All questions had higher post-scores indicating more preparedness after the intervention, except for question 2, which relates to the ability of locating the emergency contact.

**FIGURE 1:**
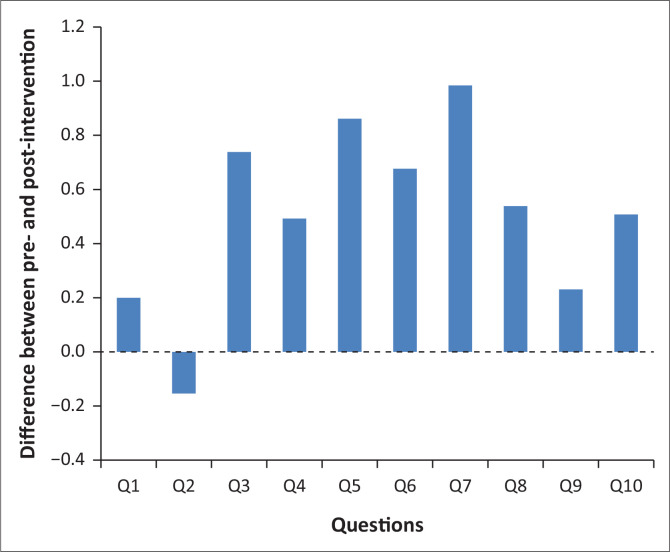
Differences between pre- and post-items for the first 10 of the 26-item audit.

#### The 10-item tool

The data in the 10-item tool excluded items Q1 and Q2 from the longer audit as well as some of the other items but retained questions mainly relating to preparation and training for COVID-19, the impact of the outbreak on self, ability to cope and manage reactions to the outbreak, and ability to manage stress levels during an upsurge of infections. According to the results, each of the 10-item showed a significant difference before and after intervention, where according to the Sum of Ranks, the post-surveys showed greater (more positive) scores than the pre-surveys.

#### Exploratory factor and reliability analysis

Regarding the EFA, all the 10-items loaded onto a single factor, which was also deemed reliable. The KMO-MSA value of 0.94 and significant Bartlett’s test of sphericity indicated that the data was appropriate for EFA. The variance extracted was also 55.31%, which is within the acceptable range. The factor loading cut-off was set to 0.40.

#### Factor scores descriptive

**FIGURE 2:**
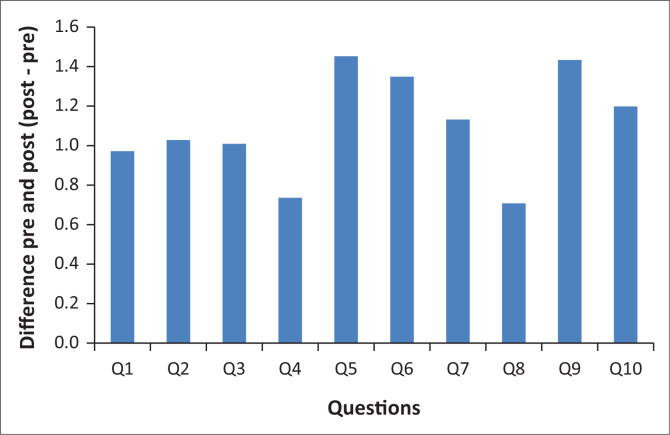
Differences between pre- and post-items for the 10-item evaluation tool.

Q10_factor_full, showed that there was a statistically significant difference pre- (*M* = 2.44, s.d. = 0.58) and post-intervention (*M* = 3.11, s.d. = 0.70) (*t* = –10.87, *df* = 159.77, *p* < 0.001). This means each factor showed a significant difference in the average factor score for pre- and post-audit data. Figure 2 shows that all the questions had higher post scores indicating more preparedness post-intervention.

### Qualitative data analysis

#### Programme feedback

The lead researcher and two research assistants tabulated and evaluated the 358 comments about the programme during four consensus meetings and grouped them into themes according to word repetition, similarities, and keywords. The three main themes that emerged made up 90.5% of the 358 feedback comments with a third category of ‘other’ added to accommodate the 34 (9.5%) remaining comments which did not fit into any specific theme. Of the 34 ‘other’ comments, eight indicated a wish for more support programmes of this nature and seven indicated it was still too early to tell the impact of the session attended. The remaining 19 were single comments that did not fit into any theme.

The findings across most of the surveyed questions confirmed significant positive change, whether it was the longer or shorter tool. This was supported by the individual feedback comments. Just over 90% of the comments described the programme as good, excellent or helpful, and indicated that it had empowered them to manage their elevated stress levels and/or support others.

## Discussion

Healthcare workers in SA came under strain because of the COVID-19 epidemic, with industrial action across the South African and other African health systems.^17,18^ This study indicates that the structured COVID-19 support programme described in this study achieved the five goals outlined when the programme was developed. These were to promote a sense of safety, a sense of calm, a sense of self– and collective efficacy, a sense of connectedness and a sense of hope. The feedback comments received from the health workers indicated an immediate benefit of feeling empowered and capable of managing their stress levels better. Other perceived benefits were an improved knowledge about the outbreak that empowered healthcare workers to cope, an enhanced ability to cope with and manage their reactions to the outbreak thus promoting self-efficacy, an enhanced ability to manage stress levels during an upsurge of infections, and an ability to recognise stress in others and help to provide support during the outbreak. This enabled them to cope with the increasing fear and anxiety that permeated the healthcare sector in SA because of the outbreak.

With regards to the evaluation tool, it was initially unclear what was going to work best, and we did not want to make the process more burdensome for healthcare workers. This is why the first part was carried out as an audit over a shorter period (six weeks) using the 26-item tool, in order to allow for review of the programme components, including the means of evaluation and feedback from the attendees. This process was useful because it enabled a review of the evaluation process when it became clear that the 26-item tool was tedious for workers already stressed by the COVID-19 situation. This gave rise to the 10-item evaluation tool, which was much shorter. Since both the 26-items and the 10-items had not been validated for use for COVID-19, it made sense to use the shorter one.

Based on the feedback received, it can be said that the model for psychological preparedness programmes for disasters can be successfully adapted to develop interventions targeted at achieving psychological preparedness for healthcare workers during the outbreak. Such interventions are likely to be beneficial in settling health workers’ sense of anxiety and fears about coping with the COVID-19 outbreak and similar epidemics.

The resource limitations at the three hospitals demanded that the programme be kept simple, relatively cheap to deliver and implementable by mental health workers with varying levels of expertise. The programme was generic enough to be used across different settings in a healthcare institution, ranging from the health administration staff, health management staff, hospital cleaners, nurses and doctors. The ability to deliver the intervention in a group format and being mindful of physical distancing, increased the capacity of the mental health teams, enabling them to reach more healthcare workers than they otherwise would have. The multidisciplinary nature of the facilitating teams also enabled the pooling of different kinds of expertise within the same session and made the sessions flow better.

### Limitations

Limitations are potential sampling bias, the descriptive nature of the study, use of an unvalidated evaluation tool to assess effectiveness of the programme, and the limited number of participants, all of which could hinder the applicability and generalisability of the study findings. The subjective nature of the feedback commentary may also be a limitation.

### Challenges

Challenges with implementation included keeping the number of attendees at the pre-agreed maximum number per session, participants turning up for sessions without booking, making the logistics difficult at times, although this was not insurmountable. Some attendees within the groups did not engage as freely as others; however, in general, even those who started off as uncommunicative would express gratitude to facilitators after the sessions.

## Conclusion

This study provides a glimpse of pre-emptive steps that could be taken to offer intervention programmes to support healthcare workers during outbreaks or pandemics. Psychological support for healthcare workers should be made an inherent part of health system responses to epidemics. Based on the positive findings in this study and the reports of high numbers of healthcare workers who experienced mental health problems during the outbreak in different parts of the world,^19,20,21,22,23^ psychological preparedness programmes should be considered to prepare healthcare workers pre-emptively to cope with the extra demands of outbreaks. The current study provides preliminary evidence that could act as a basis for developing and researching future programmes that may be useful in similar settings.

### Further research

Future research should focus on investigating accessible and effective interventions for healthcare workers to ensure that they get the support they need to help them cope during pandemics or similar stressful outbreaks. There is a possibility of expanding the intervention we have described in this study to include more healthcare workers in the EC should the number of COVID-19 infections rise again. This would also provide the opportunity to research it further.

## Figures and Tables

**FIGURE 1-A1:**
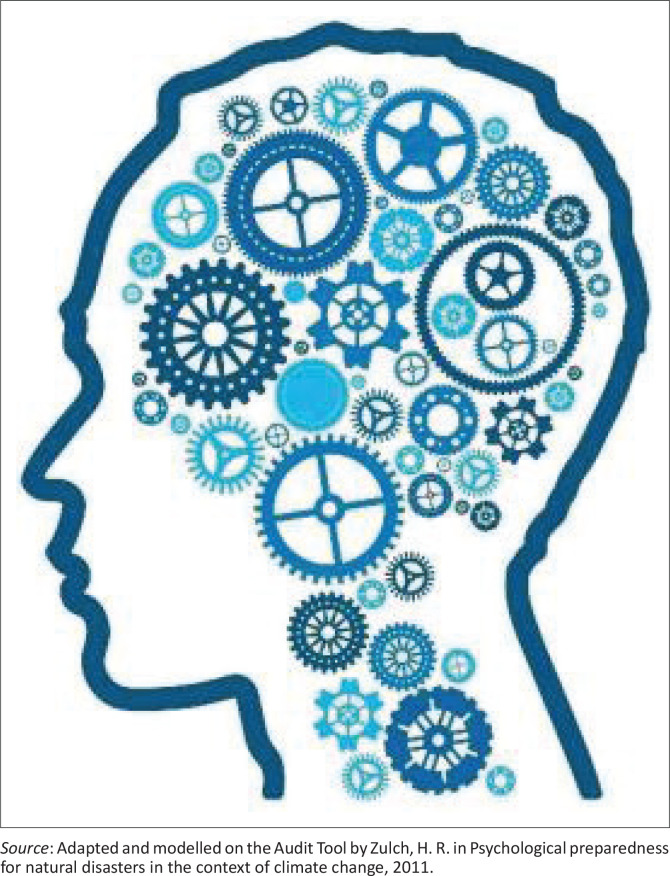
Team Sisonke: ‘Providing care for the carers. Taking steps to guard against health care worker burnout in the face of the COVID-19 Outbreak’.

## Appendix 1

### Written invitation to attend the sessions

Information for attendees
*Psychological Preparedness Training for Frontline Staff involved in the COVID-19 Outbreak*

As South Africa prepares to face the COVID-19 crisis we are faced with multiple challenges. One of these is the psychological burnout and distress related to anticipating the crisis, working during the crisis and the consequences thereafter.

Based on a psychological preparedness model from The Department of Psychiatry designed by Prof Zukiswa Zingela, supported by a multidisciplinary team, a training and intervention support program is hereby offered. It includes a focus on psychoeducation, skills to enhance team preparedness and coping skills. These sessions will be conducted in small group format and will include information that can be shared vie email to minimise face-to-face contact time.

Each group will have a maximum of 8 to 10 participants, offered on a rotational basis with strict health and safety guidelines as well as social distancing guidelines. Team leaders are asked to please identify members from various categories who may wish to attend the training and to send training requests to Head of Department of Psychiatry, Prof Z Zingela at zzingela@wsu.ac.za or via Whatsapp to … Please stick to the maximum prescribed number of attendees.

Please bring your own pen and paper for notes. The handouts and information that we would like to distribute as part of the training will be sent via email should you wish. Please supply an email address where these can be forwarded to. Group guidelines apply with regards to confidentiality and mutual respect among group members.

## Appendix 2

### 10-Item Audit Tool

**Number:** (Please use a 3-digit number sequence e.g. 001, 002, 003 etc.)

**Profession:** (Please Tick) Nurse [ ] Doctor [ ]

**Other:** ____________________

### Psychological Preparedness for COVID-19 Threat Scale (PP COVID-19)

This section is interested in how you might think, feel or respond in the face of *the COVID-19 outbreak*. Choose your answers thoughtfully and honestly.

Please respond to every statement. There are no right or wrong answers. We are interested in what *you think would be true for each statement and not what you think ‘most people’ would say or do*.

Please indicate *the extent to which each of the following statements would be true for you*.

**Table:**

		Not at all true of me	Hardly true of me	Moderately true of me	Exactly true of me
**1**	I am confident that I know what to do and what actions to take when COVID-19 hits us	□	□	□	□
**2**	I know how to adequately prepare myself to manage a case when it happens	□	□	□	□
**3**	I am familiar with COVID-19 training and materials available to me	□	□	□	□
**4**	I am knowledgeable about the impact that a COVID-19 infection can have on me and my health	□	□	□	□
**5**	I feel reasonably confident in my own ability to deal with stressful situations that I might find myself in	□	□	□	□
**6**	In a severe upsurge of COVID-19 infections I would be able to cope with my anxiety and fear	□	□	□	□
**7**	I think I am able to manage my feelings pretty well in difficult and challenging situations	□	□	□	□
**8**	When necessary, I can talk myself through challenging situations	□	□	□	□
**9**	I know which strategies I could use to calm **myself** in a severe outbreak of COVID-19	□	□	□	□
**10**	If I found myself in a severe outbreak of COVID-19 I would know how to manage my own response to the situation	□	□	□	□
Please comment on your experience during the session:					

*Source*: Adapted and modelled on the Audit Tool by Zulch, H. R. in Psychological preparedness for natural disasters in the context of climate change, 2011

## Appendix 3

### 26-Item Audit Tool

**Number:** (Please use a 3-digit number sequence e.g., 001, 002, 003 etc.)

**Profession:** (Please Tick) Nurse [ ] Doctor [ ]

**Other:** ____________________

This section is interested in how you might think, feel or respond in the face of the COVID-19 outbreak. Choose your answers thoughtfully and honestly. Please respond to every statement. There are no right or wrong answers. We are interested in what you think would be true for each statement and not what you think ‘most people’ would say or do.

Please indicate the extent to which each of the following statements would be true for you.

**Table:**

		Not at all true of me	Hardly true of me	Moderately true of me	Exactly true of me
1	I can assess the likelihood of the COVID-19 outbreak reaching us	□	□	□	□
2	I regularly monitor news bulletins re the latest news on COVID-19	□	□	□	□
3	I am confident that I know what to do and what actions to take when COVID-19 hits us	□	□	□	□
4	I would be able to locate the preparedness materials easily when I have a positive case (e.g., contact info, PPE, testing)	□	□	□	□
5	I know how to adequately prepare myself to manage a case when it happens	□	□	□	□
6	I know where I can quickly find the emergency contact when I come across a PUI/ a case	□	□	□	□
7	I am familiar with COVID-19 training and materials available to me	□	□	□	□
8	I know which personal protective measures are needed to stay safe when exposed to COVID-19 through patients care	□	□	□	□
9	I am familiar with the signs and symptoms of a COVID-19 case	□	□	□	□
10	I know what to look out for in my home and workplace if I should come across a potential COVID-19 case	□	□	□	□
11	I am familiar with the public announcements made about the COVID-outbreak and what to do for prevention	□	□	□	□
12	I know what the difference is between a need for isolation and a need for quarantine for COVID-19	□	□	□	□
13	I am knowledgeable about the impact that a COVID-19 infection can have on me	□	□	□	□
14	feel reasonably confident in my own ability to deal with stressful situations that I might find myself in	□	□	□	□
15	In a severe upsurge of COVID-19 infections I would be able to cope with my anxiety and fear	□	□	□	□
16	I think I am able to manage my feelings pretty well in difficult and challenging situations	□	□	□	□
17	When necessary, I can talk myself through challenging situations	□	□	□	□
18	seem to be able to stay cool and calm in most difficult situations	□	□	□	□
19	I know which strategies I could use to calm **myself** in a severe outbreak of COVID-19	□	□	□	□
20	If I found myself in a severe outbreak of COVID-19 I would know how to manage my own response to the situation	□	□	□	□
21	I would be able to tell easily if those/**others** around me are in distress	□	□	□	□
22	If **others** are in distress, I would know how to calm them down	□	□	□	□
23	I know which strategies I could use to calm **others** in a severe outbreak of COVID-19	□	□	□	□
24	I am able to identify my feelings pretty well in challenging situations	□	□	□	□
25	During a severe outbreak of COVID -19, I would notice if I am feeling anxious or stressed	□	□	□	□
26	usually prepare mentally for situations that might be difficult or stressful	□	□	□	□

*Source*: Adapted and modelled on the Audit Tool by Zulch, H. R. in Psychological preparedness for natural disasters in the context of climate change, 2011
